# Salivary Gland-Specific *P. berghei* Reporter Lines Enable Rapid Evaluation of Tissue-Specific Sporozoite Loads in Mosquitoes

**DOI:** 10.1371/journal.pone.0036376

**Published:** 2012-05-04

**Authors:** Chandra Ramakrishnan, Annika Rademacher, Julien Soichot, Giulia Costa, Andrew P. Waters, Chris J. Janse, Jai Ramesar, Blandine M. Franke-Fayard, Elena A. Levashina

**Affiliations:** 1 CNRS UPR9022, INSERM U963, Institut de Biologie Moléculaire et Cellulaire, Université de Strasbourg, Strasbourg, France; 2 Division of Infection and Immunity, Faculty of Biomedical Life Sciences, and Wellcome Centre for Molecular Parasitology, Glasgow Biomedical Research Centre, University of Glasgow, Glasgow, Scotland, United Kingdom; 3 Leiden Malaria Research Group, Department of Parasitology, Center for Infectious Diseases, Leiden University Medical Center, Leiden, The Netherlands; University of Heidelberg Medical School, Germany

## Abstract

Malaria is a life-threatening human infectious disease transmitted by mosquitoes. Levels of the salivary gland sporozoites (sgs), the only mosquito stage infectious to a mammalian host, represent an important cumulative index of *Plasmodium* development within a mosquito. However, current techniques of sgs quantification are laborious and imprecise. Here, transgenic *P. berghei* reporter lines that produce the green fluorescent protein fused to luciferase (GFP-LUC) specifically in sgs were generated, verified and characterised. Fluorescence microscopy confirmed the sgs stage specificity of expression of the reporter gene. The luciferase activity of the reporter lines was then exploited to establish a simple and fast biochemical assay to evaluate sgs loads in whole mosquitoes. Using this assay we successfully identified differences in sgs loads in mosquitoes silenced for genes that display opposing effects on *P. berghei* ookinete/oocyst development. It offers a new powerful tool to study infectivity of *P. berghei* to the mosquito, including analysis of vector-parasite interactions and evaluation of transmission-blocking vaccines.

## Introduction

The life cycle of *Plasmodium* takes place in a vertebrate and in an insect host. When the mosquito takes up a bloodmeal from an infected host, it ingests sexual stages of *Plasmodium*, the gametocytes. In the mosquito, the gametocytes develop into gametes which fertilise to form a zygote. The zygote develops into the motile ookinete which escapes the hostile environment of the mosquito gut by penetrating the midgut epithelium. Underneath the basal lamina, the ookinete differentiates into an oocyst which reproduces asexually to form sporozoites. These are released into the haemocoel and migrate to and invade the salivary glands. During development in the mosquito, *Plasmodium* passes through several bottlenecks of which the transition from ookinete to oocyst accounts for the greatest loss in parasite numbers [1]. The major immunity factor thioester-containing protein 1 (TEP1) together with the two leucine-rich proteins, leucine-rich repeat immune protein 1 (LRIM1) and *Anopheles Plasmodium*-responsive leucine-rich repeat 1 (APL1) are mosquito innate immunity effectors that are mainly responsible for the losses at this stage [2], [3], [4]. Significant parasite loss also occurs during the following stage where ‘midgut sporozoites’ (mgs) are released from the oocysts into the haemocoel and colonise the salivary glands although the mechanisms and effector molecules that invoke such losses remain obscure. Only ∼20% of mgs have been shown to invade salivary glands [5]. Although phagocytosis of sporozoites in the haemocoel has been reported in *Anopheles* and *Aedes* mosquitoes [5], [6], [7], the fraction of sporozoites eliminated by phagocytosis is small despite the capability of haemocytes to phagocytose large number of foreign particles or bacteria [5]. Some of the sporozoites erroneously locate and become trapped in distal extremities irrelevant for the transmission cycle such as wings or legs [5]. At the molecular level, the mosquito serine protease inhibitor (serpin) 6 (SRPN6) has been shown to be implicated in reducing the numbers of salivary gland sporozoites (sgs) [8], however the precise mechanism of reduction by SRPN6 remains to be uncovered. As sgs are responsible for the establishment of an infection in the vertebrate host, new methods are needed to dissect mechanisms that affect sgs numbers in the mosquitoes.

A series of methods have been developed which vary in accuracy, sensitivity and simplicity, nevertheless quantification of *Plasmodium* loads in mosquitoes remains a laborious and time-consuming task. Either direct observation and counting of parasite forms [9], [10], [11], [12], [13] or quantification of parasite components [14], [15], [16] have been employed but all suffer from significant technical difficulties. Here, we report the development of a biochemical assay to evaluate parasite loads in salivary glands of infected mosquitoes that avoids dissection of salivary glands and isolation of sporozoites. The assay uses transgenic *P. berghei* designed to express a reporter gene exclusively in sgs and not mgs. Mining the results of a subtractive hybridisation screen for genes that expressed in *P. berghei* mgs or sgs [17] and proteome analyses of *P. falciparum* mgs or sgs [18] led to a choice of three promoters to drive expression of the reporter gene: upregulated in infective sporozoites 3 (*uis3*), *uis10* and *glyceraldehyde-3-phospho-dehydrogenase* (*glyc*). *P. berghei* expressing a fusion protein of GFP and luciferase (GFP-LUC) have been used to detect blood and liver stages of the parasite as well as to visualise infection in dissected organs or whole bodies of mice [19], [20], [21]. Luciferase activity of such reporter lines has been previously exploited for screening of antimalarial drugs [22]. We chose *gfp-luc* as a fusion reporter gene enabling parasite detection by fluorescence microscopy and enzymatic activity measurement of luciferase in the transgenic lines. We generated and characterised two salivary gland-specific reporter *P. berghei* lines and established a simple biochemical assay to examine sgs loads. The efficacy of the assay was shown in experiments in which sgs loads were determined in mosquitoes after down regulation of known immune effector molecules.

## Results

### Generation and Molecular Analyses of Transgenic Reporter Parasite Lines

**Figure 1:**
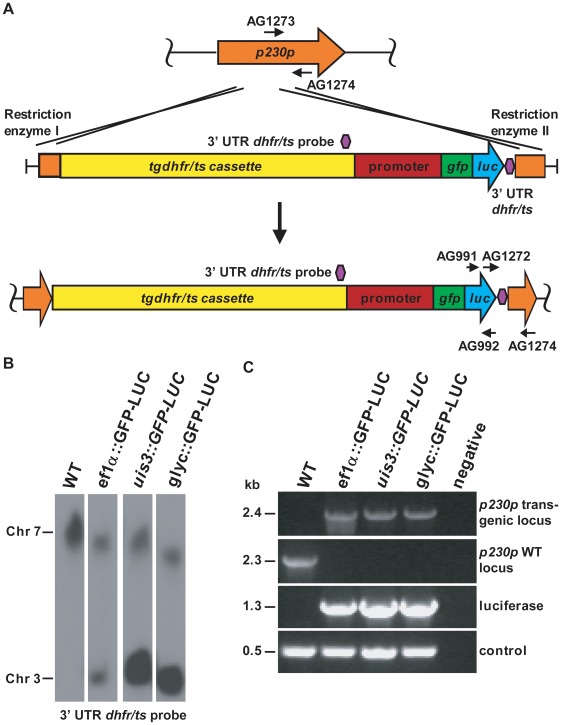
Generation of transgenic parasites expressing *gfp-luciferase* under salivary gland-specific promoters. (A) Schematic representation of a strategy used to obtain transgenic parasites. The top panel shows the wild type locus of *p230p*; arrows indicate the primers used to identify an intact *p230p* locus. The middle panel shows the linearised plasmid containing the 5′ and 3′ *p230p* fragments used for recombination (orange), *tgdhfr/ts* flanked by the 5′ UTR and 3′ UTR of *pbdhfr* (*tgdhfr/ts* cassette, yellow), the salivary gland-specific promoter (magenta), the *GFP-luciferase* fusion gene (green and blue) and a second *pbdhfr* 3′ UTR (dark blue). Crossing-over event is illustrated by the two crosses. The bottom panel shows the resulting disrupted, transgenic locus of *p230p*. Arrows represent the primers used for the identification of the disrupted *p230p* locus or of *luciferase*. Blue blocks illustrate the probes used for the FIGE analysis. (B) Southern analysis of FIGE separated chromosome of cloned transgenic lines confirms correct integration of the construct into the *230p* locus on chromosome 3. Hybridisation with the 3′UTR *dhfr/ts* probe recognises the integrated construct in chromosome 3 and the endogenous *P. berghei dhfr/ts* gene on chromosome 7. Note the more intense signal of chromosome 3 in the transgenic lines resulting from the two 3′UTR *dhfr/ts* regions in the construct. (C) Diagnostic PCR analyses of cloned transgenic parasites, confirming the correct integration of the constructs.

*Gfp-luc* (*gfp* mutant 3 and firefly *luciferase IAV* [21]) fusion genes under the control of the salivary gland-specific promoters *uis3* (PBANKA_140080), *uis10* (PBANKA_112810 ) or *glyceraldehyde-3-phospho-dehydrogenase* (*glyc,* PBANKA_132640 ) were cloned into a plasmid containing the *Toxoplasma gondii dihydrofolate reductase/thymidylate synthase* (*tgdhfr/ts*) gene (selectable marker for pyrimethamine resistance) flanked by the 5′ and 3′ UTRs of the *pbdhfr* gene. For the *uis3*, *uis10* and *glyc* promoters, fragments of 1895 bp, 1824 bp and 1914 bp, respectively, were used to generate the constructs. It has been previously observed that transgenic parasites producing the fusion protein GFP-LUC were considerably less fluorescent compared to a parasite lines expressing *GFP* alone [9], possibly due to steric hindrance of the fluorophore. Therefore in the *uis3::GFP-LUC* line, the linker between *GFP* and *luciferase* was changed from Gly-Ile-Leu-Ala-Ser to Gly-Gly-Pro-Ser-Gly, allowing more flexibility between GFP and luciferase. The DNA vector (derived from pAMA1RFP230p [pL1157]) also contained two flanking arms homologous to the *p230p* gene which served to introduce the transgene into the genome and stably disrupt the non-essential *p230p* locus [23] by homologous recombination (Figure 1A). The resulting plasmids pL1327 (*uis3::gfp-luc*), pL1163 (*uis10::gfp-luc*) and pL1171 (*glyc::gfp-luc*) were then linearised and individually transfected into *P. berghei* ANKA cl15cy1 parasites as described [10]. Selection of transgenic, pyrimethamine resistant parasites yielded the following three transgenic reporter lines: *uis3*::*GFP-LUC*, *uis10*::*GFP-LUC* and *glyc*::*GFP-LUC*.

These uncloned lines were maintained in mice under pyrimethamine drug pressure and fed to *A. gambiae* G3 mosquitoes. At 7 days post infection (dpi) oocysts were analysed for GFP expression. No GFP signal above background was detected in oocysts of *uis3::GFP-LUC* and *glyc*::*GFP-LUC*. However, GFP was detected in oocysts of *uis10*::*GFP-LUC* (data not shown) and therefore, this line was excluded from further analysis.

Before proceeding with the analysis of the *uis3::GFP-LUC* and *glyc*::*GFP-LUC* lines, these parasites were cloned twice by limiting dilution cloning. Correct integration of the transgenes into the *p230p* locus situated on chromosome 3 [24] was verified by Southern blotting analysis of field inverted gel electrophoresis (FIGE) separated chromosomes (Figure 1B) and confirmed by PCR (Figure 1C). A cloned parasite line, *ef1α*::*GFP-LUC*, that expresses the *GFP-LUC* fusion gene constitutively [21], was used as a reference line and provided an additional control in the molecular analysis. The cloned lines selected for further characterization were *uis3::GFP-LUC* clone 4.4 (thereafter referred to as *uis3*::*GFP-LUC*) and *glyc*::*GFP-LUC* clone 2.5 (thereafter referred to as *glyc*::*GFP-LUC*).

### Expression of *uis3*::*GFP-LUC* and *glyc*::*GFP-LUC* Reporters during the Parasite Life Cycle

**Figure 2:**
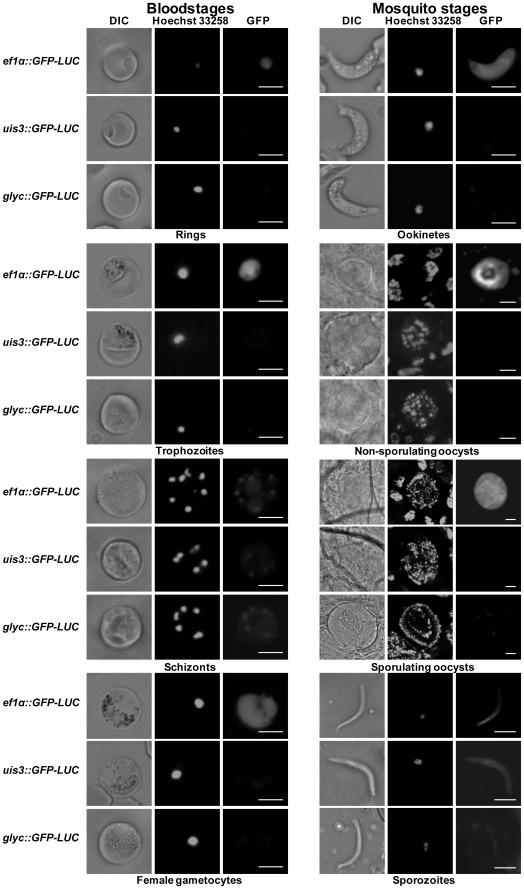
GFP detection in blood and mosquito stages by fluorescence analysis. (A) For blood stage images, blood of infected mice was diluted and stained with the blue nuclear dye Hoechst 33258. Ookinetes were cultured, pelleted and stained with Hoechst 33258 before imaging. To image oocysts and salivary gland sporozoites, mosquitoes were infected with the transgenic parasites. Midguts containing oocysts were dissected directly into PBS/Hoechst 33258 11–21 dpi; infected salivary glands were dissected into PBS/Hoechst 33258 16–21 dpi. Oocysts and sporozoites were stained with the nuclear dye for 15–30 min. The GFP was visualized using the GFP fluorescence channel, the nuclei using the UV channel. The scale bars represent 5 µm for all the blood stages, ookinetes and sporozoites and 10 µm for oocysts.

To verify the specificity of the *uis3* and *glyc* promoters, the blood and mosquito stages of *uis3::GFP-LUC* and *glyc*::*GFP-LUC* lines were examined throughout the life cycle by live fluorescence microscopy using the *ef1α*::*GFP-LUC* line as a control (Figure 2).

In blood stage ring-forms, trophozoites, and gametocytes, no GFP signal above background was observed for *uis3::GFP-LUC* and *glyc*::*GFP-LUC.* As expected, GFP expression detected in all blood stages of *ef1α*::*GFP-LUC* (Figure 2). A faint but distinct GFP signal was observed in developing schizonts of both the *uis3::GFP-LUC* and *glyc*::*GFP-LUC* lines. The bloodstages are known to use glycolysis; therefore, the observed GFP signal in the *glyc*::*GFP-LUC* line is not surprising. However, *uis3* (and *uis10*) have not been previously shown to be expressed in bloodstages, but only in sgs [17].

**Figure 3:**
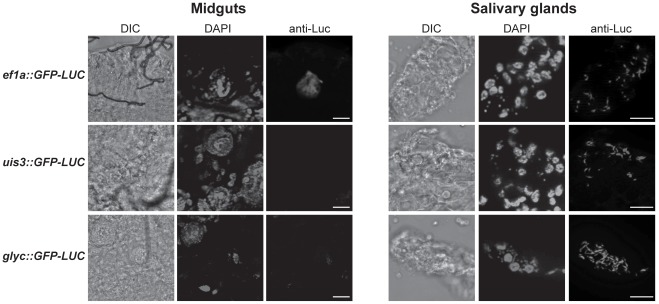
Luciferase detection in mosquito stages by immuno-fluorescence assay (IFA). Luciferase expression in mgs and sgs was detected by immunofluorescence in dissected fixed midguts and salivary glands (17–21 days post infection) using anti-firefly luciferase antibody. Nuclei were visualized with DAPI. The scale bars represent 20 µm.

Cultured ookinetes generated no GFP signal. No GFP-fluorescence was detected in non-sporulating or sporulating oocysts at 11 and 21 dpi for *uis3::GFP-LUC* and *glyc*::*GFP-LUC*. To confirm these results, immunofluorescence assays (IFA) of mgs of *uis3::GFP-LUC* and *glyc*::*GFP-LUC* using anti-luciferase antibody were performed and compared to *ef1α*::*GFP-LUC*. No signals could be observed for the *uis3::GFP-LUC*. Low level of *glyc*::*GFP-LUC* expression was detected in 10% of mgs, whilst mgs of *ef1α*::*GFP-LUC* were readily detected (Figure 3). Between 16 and 21 dpi, salivary glands from infected mosquitoes were dissected and GFP fluorescence in sporozoites was analysed by microscopy. Sporozoites of the three transgenic lines showed varying intensities of fluorescence: the GFP-intensity of sporozoites of *uis3::GFP-LUC* and *glyc*::*GFP-LUC* was considerably lower than those of *ef1α*::*GFP-LUC* (Figure 2). Further IFA using the anti-luciferase antibody revealed strong expression of the reporter in sgs of all three transgenic lines, demonstrating that the highest levels of *glyc* and *uis3* expression occurred in the salivary gland stages of *P. berghei* (Figure 3).

### Levels of Bioluminescent Signal of Salivary Gland Sporozoites are Proportional to the Sporozoite Numbers

**Figure 4:**
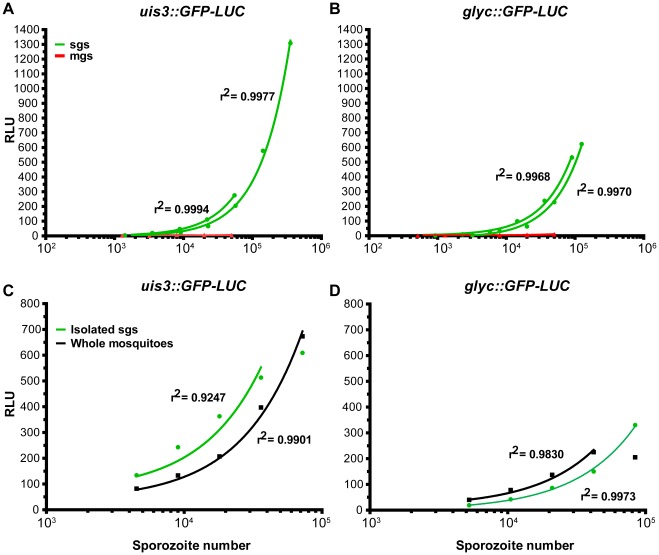
Correlation between sporozoite numbers and luciferase activity in whole mosquitoes, and dissected midgut and salivary gland sporozoites of *uis3::GFP-LUC* and *glyc::GFP-LUC*. Mosquitoes were infected with either *uis3::GFP-LUC* (A and C) or *glyc::GFP-LUC* (B and D) and sporozoites extracted from salivary glands (sgs), midguts (mgs) or whole mosquitoes were collected 18–19 dpi. The sporozoites and whole mosquitoes were lysed and dilution series of cell extracts were generated and used to perform luciferase assays. Luciferase activity was measured and plotted as the value after subtraction of the baseline against the sporozoite number (RLU). Goodness of the linear curve fit is given as r^2^. Graphs representing two independent experiments are shown. 10^4^ sgs correspond to 1.1 mosquito equivalent in (C) and 1.2 in (D). Outliers have been omitted from the curve fit (i.e. highest sgs concentrations for isolated sgs for (C) and whole mosquitoes (D)).

We next examined whether the luciferase reporter could be used for sgs quantification. To this end, a standard curve was generated to identify the correlation between bioluminescent signals and sgs numbers for both the *uis3::GFP-LUC* and *glyc*::*GFP-LUC* parasite lines. Sgs were isolated from 40–60 mosquitoes by salivary gland dissection and the sporozoite numbers were counted by haemocytometry. After the counting the sporozoites were lysed and a dilution series was generated. Cell extracts were then used to perform a luciferase assay. Linear correlations between the sporozoite numbers and luciferase signal intensity (RLU) were observed for both lines in a range from approximately 10^3^ to 10^5^ sgs (detection threshold 10^3^–10^4^) (Figure 4A, B). Sporozoites isolated from dissected midguts treated in the same fashion did not display any luciferase activity above background levels (Figure 4A, B). To establish if there was a correlation between the intensity of the bioluminescent signals of the isolated sporozoites and the sporozoites present in the whole mosquitoes, a standard curve was generated using *A. gambiae* infected with *uis3::GFP-LUC* or separately, with *glyc::GFP-LUC* (Figure 4C, D, Figure S1). The mosquitoes were then separated into two groups: as above, salivary glands were dissected from the first group and the isolated sgs were subjected to a luciferase assay. The other group was used to perform the luciferase assay using whole mosquitoes. For both groups, the dilution series were performed in the same fashion to obtain a standard curve. The luminescence signal of the isolated sgs and sgs in whole mosquitoes followed a linear curve with the same detection threshold of 10^3^ sgs as in the experiments described above (Figure 4C, D, Figure S1). Our results demonstrate that the bioluminescent signal of isolated sgs correlates to the signal produced by infected whole mosquitoes. On rare occasions we observed that some readings at the higher concentration of isolated and whole mosquito extracts produced aberrant data (Figure 4C, D, Figure S1), these data points were excluded from curve fit.

Taken together our results show that our reporter lines are specific for sgs and can be used in whole mosquito samples to quantify sgs loads. The specific expression pattern of the reporter genes eliminates the time-consuming dissection of the salivary glands; instead whole mosquitoes can be used to evaluate sporozoite numbers.

### Evaluation of Sporozoites in Salivary Glands of Mosquitoes Depleted for Major Proteins Involved in the Control of *P. berghei* Development

A fair correlation between numbers of human hepatocyte cultures infected with *luciferase*-expressing *P. berghei* sporozoites and measured luminescence has previously been reported [21]. Similarly, oocyst numbers of ef1*α*::*GFP-LUC* determined by fluorescent microscopy were shown to correlate to luciferase activity (data not shown). We therefore examined whether our salivary gland-specific reporter line *glyc*::*GFP-LUC* could be exploited to quantify parasite loads in the mosquitoes with modulated expression of two major regulators of *P. berghei* development. As *uis3::GFP-LUC* and glyc::GFP-LUC displayed specificity to sgs and similar fluorescence intensity, only one line, *glyc*::*GFP-LUC*, was further used in these studies.

**Figure 5:**
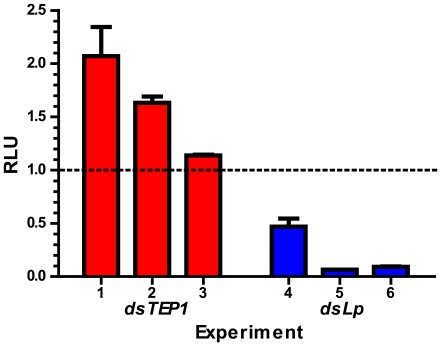
Relative luciferase activity in *TEP1* and *Lp* knockdown mosquitoes infected with *glyc::GFP-LUC*. Mosquitoes were injected with dsRNA prior to infection with *glyc::GFP-LUC*. Surviving mosquitoes 18–21 dpi were freeze dried, ground and the luciferase activity was measured. The values are normalised to the values in control treatment (*dsLacZ*). Shown are the results of 3 independent experiments for each treatment expressed as means of duplicate or triplicate measurements; error bars represent the standard error of the mean. The dotted line marks the value 1, corresponding to the respective controls. Sample sizes in experiment 1: *dsLacZ* (n = 21), *dsTEP1* (n** = **17); experiment 2: *dsLacZ* (n = 19), *dsTEP1* (n** = **17); experiment 3: *dsLacZ* (n = 26), *dsTEP1* (n** = **11); experiment 4: *dsLacZ* (n = 21), *dsLp* (n* = *20); experiment 5: *dsLacZ* (n = 26), *dsLp* (n = 8); experiment 6: *dsLacZ* (n = 44), *dsLp* (n = 39).

Mosquitoes were injected with either double-stranded RNA against the negative regulator of ookinete development *TEP1* (*dsTEP1*), the positive regulator of ookinete development lipophorin (*dsLp*) or a bacterial *LacZ* gene (*dsLacZ*) as a control. Injected mosquitoes were allowed to recover for four days, infected with *glyc*::*GFP-LUC*, collected at 18–21 dpi and kept frozen at −20°C until further analysis. As a negative control, mosquitoes infected with the PbGFP_CON_ which expresses *GFP* but not *luciferase* or uninfected mosquitoes were used. Pooling mosquitoes of the same experimental group enabled us to obtain an average reading for all mosquitoes from one condition. Whole mosquito lysates were cleared by centrifugation and used to determine the luciferase activity. Three independent experiments were performed using mosquitoes treated with either *dsTEP1* or *dsLp* and the luciferase activity was measured and compared to *dsLacZ* (Figure 5). Silencing of *TEP1* resulted in a moderate but significant increase in sporozoite levels; this up to two-fold increase is consistent with the previously reported effect on oocyst numbers upon *TEP1* knockdown where two- to five-fold increase in oocyst loads were reported [2], [25], [26]. Depletion of lipophorin resulted in a two- to ten-fold decrease of luciferase activity (Figure 5). The levels of luciferase activity were comparable to background levels, i.e. levels of negative controls with PbGFP_CON_ infected or uninfected mosquitoes and lysis buffer only (data not shown). A two-fold reduction of oocyst numbers and their rates of development in the mosquitoes silenced for *Lp* has been previously reported [27], [28]. To distinguish between complete and partial block in sgs development in *Lp*-silenced mosquitoes, we quantified PbGFP_CON_ parasite numbers at 19–21 dpi by microscopic analysis. A dramatic reduction of on average 77% in the sporozoite load was observed in *dsLp*-injected mosquitoes both in salivary gland sporozoites and in midgut sporozoites, whereas an up to five-fold increase in sgs was detected in mosquitoes silenced for *TEP1* (Table S1). Our data imply that the new assay is a sensitive method to detect differences in sgs loads. However infections with low sgs loads (i.e. below 10^3^ sporozoites) would yield a bioluminescent signal below the threshold value of negative controls and such infections would require further microscopic analysis. Our results further identify Lp as the first mosquito factor that is required for optimal sporozoite development and completion of *P. berghei* life cycle within the insect vector.

## Discussion

We have generated two clonal transgenic *P. berghei* lines which express a fusion *GFP-LUC* gene specifically in salivary gland sporozoites. The erythrocytic and mosquito stages of *uis3::GFP-LUC* or *glyc*::*GFP-LUC* have been examined by fluorescent microscopy and the reporter genes have been found to be expressed at the schizont and the salivary gland sporozoite stages only. The detected activity of both promoters at the schizont stage is unexpected and will require further investigation. Importantly for this study, only salivary gland sporozoites of all the mosquito stages expressed the reporter gene under the control of *UIS3* and *Glyc* promoters; therefore quantification of the reporter activity in the whole mosquitoes directly corresponds to the levels of infective sporozoites.

It is known that the fusion protein GFP-luciferase generates lower GFP-fluorescence intensity in transgenic *Plasmodium* parasites compared to GFP (using the same promoter elements). We have tested the effect of modification of the linker sequence between the *GFP* and *luciferase* open reading frames in *uis3::GFP-LUC* parasites but unfortunately we did not observe a significant increase in GFP signals. These results suggest that a different strategy based on integration of two independent expression cassettes for *luc* and *GFP* should be used to improve the sensitivity of the approach.

Here, we have established a simple biochemical assay to estimate numbers of sgs in mosquitoes for which no signal above background can be observed for mgs. Therefore, this method represents a major step towards simplification of sgs evaluation as no dissections are required, and whole mosquitoes can be used for the assay. A caveat of this method lies in the detection threshold of the bioluminescent signal: the standard curves illustrated in Figure 4 (and Figure S1) show that the detection threshold generally lies above 10^3^ sgs. Experiments on a single mosquito and on small pools of infected mosquitoes yielded no detectable luminescence signal (data not shown). Here we demonstrate that this limitation can be overcome by pooling together all mosquitoes from a given experimental group therefore providing an average reading of parasite levels for a given sample. As a proof of principle, we examined sporozoite development in mosquitoes silenced for two key genes that regulate parasite development at the ookinete/oocyst stage. Here we show that *TEP1* knockdown results in higher sporozoite infections, although differences in sporozoite loads are less pronounced than at the oocyst stage. Depletion of the major yolk protein lipophorin previously shown to dramatically inhibit growth and development of oocysts [27], reduces sporozoite levels by 80% but does not completely abort sgs development. We conclude that the luciferase-based quantification developed here represents a simple and rapid method to evaluate sgs loads which can be used in high throughput screening of mosquitoes for *Plasmodium*-resistance genes. Such a screening should detect genes involved at regulation of all stages of parasite development, from midgut to salivary gland invasion, a topic which to date has not been studied in detail. We believe that this method will be of use not only for vector biologists and for studies on *Plasmodium* biology but also for evaluation of transmission-blocking vaccines.

## Materials and Methods

### Generation of Transgenic Parasites

Primers 2601 (5′-CAAGATAGAAGAAGCCGTTCACAAGCC-3′) and 2602 (5′-GTACCGATATCCCCGGGCTGCA-3′) were used to amplify *GFP* from plasmid pL0031 [29] and were cloned into an intermediate vector between the promoter of *P. berghei* elongation factor (*pbef*) 1α and the 3′ untranslated region (UTR) of *pbdhfr* using *Bam*HI and *Xba*I. The *GFP* and the *P. berghei dihydrofolate reductase* (*pbdhfr*) 3′ UTR were excised together using *Bam*HI and *Kpn*I and inserted into another intermediate vector where the insert was flanked by the *AMA1* promoter and by a fragment of *p230p* located in the 3′ region of *p230p*. The plasmid contained also an ampicillin resistance gene, a 5′ fragment of *p230p* and the *tgdhfr/ts* gene flanked by 5′ and 3′ UTRs of *pbdhfr*. The *UIS10* promoter was amplified by primers 2603.

(5′-CGGATATCGCGGCCGCGAGTATAGGATAGATAATTTTTTTTGTGG-3′) and 2604 (5′CGGGATCCCCATGGTCTTTCACATTTACGCCAATAATTTTTTTAATG-3′). The *Eco*RV/*Bam*HI digested PCR product was then inserted into the digested vector. After an *Hpa*I/*Kpn*I digest, the luciferase gene was inserted into the vector to yield pL1163. The *UIS10* promoter could be exchanged with the *UIS3* or *Glyc* promoters, which were amplified with primers 2605 (5′-ATAAGAATGCGGCCGCGGACATATTTTGGGACTATCCAGGTATAGTGTG -3′) and 2606 (5′-CATGCCATGGATATTTGTTATTTGTCTAAATAATGC-3′) or 2607 (5′-ATAAGAATGCGGCCGCGTATAAACTGAGTTAAGGGAAGTGG-3′) and 2608 (5′-CATGCCATGGTTTTATGTTTTTTTAAAATATTATATTGCTTG-3′), respectively. *Not*I/*Nco*I-digested fragments were used for replacement of the promoter yielding pL1171 for the *Glyc* promoter. The linker between *GFP* and *luciferase* was exchanged in the vector carrying the *UIS3* promoter by using AG465 (5′-CGGGATCCGGAGGACCATCAGGAATGGAAGACGCCAAAAACa-3′) which annealed to the 3′ end of *GFP* and AG466 (5′- ACCCAGTAGATCCAGAGGAATTCATTATCAGT-3′) annealing downstream of the start codon to amplify the linker region. The PCR product was excised, ligated upstream to the *UIS3* promoter fragment and downstream to the luciferase gene and cloned into the *UIS3* intermediate construct lacking *gfp* described above. The *GFP* gene was then re-inserted using the *Bam*HI restriction site to yield pL1327.

pL1163 and pL1171 were linearised with *Sac*II and pL1327 with *Spe*I and *Bgl*I before transfection into *P. berghei* ANKA cl15cy1 [10] using the standard protocol by Janse *et al*. [10]. After two rounds of drug selection in mice using 70 mg/l of pyrimethamine in drinking water, *uis3::GFP-LUC* and *glyc::GFP-LUC* were cloned by two rounds of limiting dilution cloning to yield the strains *uis3::GFP-LUC* clone 4.4 and *glyc::GFP-LUC* clone 2.5.

### Molecular Analyses of Transgenic Parasites

For field inverted gel electrophoresis (FIGE) analysis, infected blood was pelleted and lysed for 3–5 min on ice in red blood cell lysis buffer (150 mM NaCl, 10 mM KHCO_3_, 1 mM EDTA, pH 7.4). The pelleted parasites were then resuspended in an equal volume of 1.5–2.0% low melting agarose/TES (50 mM Tris-HCl, 100 mM NaCl, 5 mM EDTA, pH 8.0). These agarose blocks were then treated with 10 µg/ml Proteinase K/SE buffer (500 mM EDTA, 1% N-lauroylsarcosine, pH 8.0) at 37°C overnight and then transferred into SE buffer. Chromosomes were then separated using FIGE, depurinated in 250 mM HCl for 15 min and denatured for 20–30 min in 0.5 M NaOH, 1.5 M NaCl. The gel was then neutralised in 20×SSC (3 M NaCl, 300 mM sodium citrate) and the DNA was transferred on to Hybond-N+ (GE Healthcare) nylon membrane and cross-linked using standard procedures [30].

The probe template was amplified using L692 (5′-CTTATATATTTATACCAATTG-3′) and L693 (5′-GTTTTTTTTTAATTTTTCAAC-3′). In 16 µl total volume, 80–100 ng of probe template and 10 ng of hexanucleotides from the hexanucleotide reaction mix (Roche) were boiled for 5–10 minutes at 100°C and kept on ice. Then, 2.5 nmol each of dCTP, dGTP and dTTP, 30 µCi α-^32^P-dATP (10 µCi/µl, GE Healthcare) and 2 units Klenow polymerase ((2 U/µl, Roche) were added and incubated for 30 min at 37°C to obtain labelled double stranded probes. Labelled probes were purified using Micro Bio-Spin P-30 Tris Chromatography Columns (Biorad) according to the manufacturer’s protocol, and then denatured for 5 min at 95°C. The membrane was pre-hybridised for 1 h at 65°C before the probe was added. After overnight hybridisation to the probe, the blot was washed twice with 3×SSC/0.5% (v/v) sodium dodecyl sulphate (SDS) and once with 1×SSC/0.5% (v/v) SDS and exposed to X-ray film.

Diagnostic PCRs were performed using the primer combinations AG991 (5′- GATACGCCCTGGTTCCTGG-3′) and AG992 (5′-GTCGGGAAGACCTGCCAC-3′) to detect *luciferase* and AG1272 (5′-GAGCACGGAAAGACGATGAC -3′) or AG1273 (5′-GAAAGGATGGTACTAAAATAGATGGATGC-3′) together with AG1274 (5′- CCAACTACATCATTTTCTATGGCCTC-3′) to detect the *p230p* transgenic or wild type locus respectively. As a positive control, the unrelated, the *P. berghei guanylyl cyclase β* gene was amplified using AG975 (5′-TGAAGGAAACAGATAAAATAAAGAG-3′) and AG976 (5′-GTAAACGATAACTGCGTCAAGTG-3′).

### Mosquito Rearing and Parasite Maintenance

*A. gambiae* mosquitoes, G3 and Ngousso [31] strains, were reared at 28°C and 70–80% humidity under 12 h/12 h light/dark cycle and maintained using 10% sucrose. Parasites were maintained in CD1 mice. Anaesthetised mice infected with the different parasite strains were used for mosquito infections. Parasitaemia and gametocytaemia of mice were estimated using thin smears of tail blood stained with Diff-Quick I- and II- (eosin G and thiazine dye, Dade Behring). Mosquitoes infected with *P. berghei* were maintained at 20°C.

### Imaging

Imaging was performed using the Axiovert 200 M fluorescence microscope (Zeiss) together with the Axiovision version 4.7 software. Raw images were then processed using ImageJ version 1.43 u or 1.45 s.

To image blood stages, a droplet of tail blood was diluted in PBS containing 10 µg/ml Hoechst 33258 (Invitrogen) nuclear dye. Exposure time for the GFP channel was 2 s. Ookinetes were cultured by using the blood of mice which had received a blood passage of 5×10^7^ parasites 3 days before. The blood was suspended in ookinete medium (RPMI1640, 1.75 g/l NaHCO_3_, 50 mg/l hypoxanthine, 100 µM xanthurenic acid, pH 7.4)/20% FBS and cultured at 20°C for 24 h. The parasites together with the blood cells were pelleted at 500 g and resuspended in PBS/10 µg/ml Hoechst 33258. For the GFP channel, 3 s exposure time was used. To image oocysts, infected mosquitoes were dissected in PBS/10 µg/ml Hoechst 33258 and midguts were left to stain for 15–30 min at RT. Oocysts dissected between day 11 and 21 were exposed to the GFP channel for 2.5 s. To image salivary gland sporozoites, salivary glands of infected mosquitoes were dissected 16–21 dpi into PBS/10 µg/ml Hoechst 33258, mounted, left for 15 min at RT and exposed to the GFP channel for 3 s.

### Immunostainings

Dissected midguts and salivary glands (17–21 days post infection) were fixed for 30 min in ice-cold 4% paraformaldehyde, washed three times in PBS, blocked for 1 h (1% BSA, 0.1% Triton X-100 in PBS), and incubated overnight at 4°C with anti-firefly luciferase antibody (rabbit polyclonal ab21176, Abcam, dilution 1∶800), followed by an incubation with the secondary goat anti-rabbit Cy3 antibody (Molecular Probes, dilution 1∶1000) and DAPI (Molecular Probes, 1 µg/mL). Samples were mounted using Aqua-Poly/Mount (Polyscience). All samples were examined with Axiovert 200 M fluorescence microscope equipped with an ApoTome slider module (63×objective, Zeiss) and images were then processed using ImageJ version 1.45 s.

### RNAi Gene Silencing in Mosquitoes

The plasmids pLL100 [2], pLL17 [2] and pLL345 [27] carrying gene fragments of *β-galactosidase* (*lacZ* ), *A. gambiae TEP1* and *Lp* [27], [32] respectively flanked by two T7 promoters were linearised before single stranded RNA (ssRNA) was synthesised with the T7 MEGAscript kit (Ambion) using the manufacturer’s instructions. Complementary ssRNAs were then annealed by placing in boiling water which was left to cool to RT. Mosquitoes were then injected with 69 nl of double stranded RNA (dsRNA) 2–4 days prior to infection.

### Isolation of sgs and Luciferase Assay

At 18–21 dpi, *A. gambiae* G3 mosquitoes were either frozen at −20°C or in liquid nitrogen if used as whole mosquitoes or drowned in 70% ethanol and rinsed three times with PBS before isolation of sgs. Midguts and salivary glands were dissected out into PBS and pooled respectively. For standard curved comparing isolated sgs and whole mosquitoes, mosquito pools were halved and processed either by drowning the whole mosquitoes or isolating the sgs. Midguts and salivary glands were then separately ground in PBS using a pestle and tissue debris was removed by passing the suspensions first through 70 µm and subsequently 40 µm cell strainers. Sporozoite numbers were estimated using a haemacytometer (Thoma Cell) and a dilution series was generated. To 90 µl of sporozoite suspension (or PBS as a baseline), 22.5 µl Cell Culture Lysis Reagent (Luciferase Assay System, Promega) was added. For gene knockdown experiments, infected mosquitoes on day 18 or 21 were frozen at −20°C as a pool. They were then freeze dried in liquid nitrogen, ground in a Retsch MM300 grinder using a steel ball for 1 min at 20 rotations/s. The freezing and grinding step was repeated. Ground mosquitoes were resuspended in 40 µl of 1∶5 diluted lysis reagent per mosquito. Lysis was performed for 20 min at RT. For gene knockdown experiments, uninfected mosquitoes, mosquitoes infected with PbGFP_CON_ and/or lysis buffer alone were used as negative controls. Luciferase activity was measured after adding 100 µl Luciferase Assay Reagent in Luciferase Assay Buffer (Luciferase Assay System, Promega) to 80 µl lysate in relative light units (RLU) using the Bertholds Mithras LB940 luminometer and the MicroWin 2000 version 4.37 software. For the standard curves, the baseline was measured at least in a duplicate and the average values were used for correction. Curves were fitted using linear regression. Obvious outliers were omitted from the curve fit. For gene knockdown studies, luciferase activity was measured in triplicates and values were normalised to the mean value of the background measurements that were normalized to the corresponding values of *dsLacZ* controls.

## Supporting Information

Figure S1 **Correlation between sporozoite numbers and luciferase activity in sgs and in whole mosquitoes of** ***uis3::GFP-LUC*** **and** ***glyc::GFP-LUC*** **.** Mosquitoes were infected with either *uis3::GFP-LUC* (A and B) or *glyc::GFP-LUC* (C and D) and sporozoites extracted from salivary glands or whole mosquitoes from the same experiment were collected 18–19 dpi. The sporozoites and whole mosquitoes were lysed and dilution series of cell extracts were generated and used to perform luciferase assays. Luciferase activity was measured and plotted as the value after subtraction of the baseline against the sporozoite number (RLU). Goodness of the linear curve fit is given as r^2^. 10^4^ sgs correspond to 3.1 mosquito equivalents (A), 2.2 in (B), 6 in (C) and 2.1 in (D). In (A) reading of the highest sgs concentration for whole mosquitoes was excluded from the curve fit as an outlier.
(TIF)

Table S1 **Sporozoite numbers in** ***dsTEP1*** **and** ***dsLp*** **mosquitoes.** *A. gambiae* mosquitoes injected with dsRNA against *TEP1*(*dsTEP1*), *Lp* (*dsLp*) and *LacZ* (*dsLacZ*, control) were infected with PbGFP_CON_ and salivary gland sporozoites were isolated from 7–18 mosquitoes 19–21 dpi to quantify sporozoite loads.
(DOC)
