# Supplementary figures and images for: Salivary Gland-Specific P. berghei Reporter Lines Enable Rapid Evaluation of Tissue-Specific Sporozoite Loads in Mosquitoes
Source: PLoS One. 2012 May 4;7(5):e36376. doi: 10.1371/journal.pone.0036376 (PMC3344870; doi:10.1371/journal.pone.0036376)

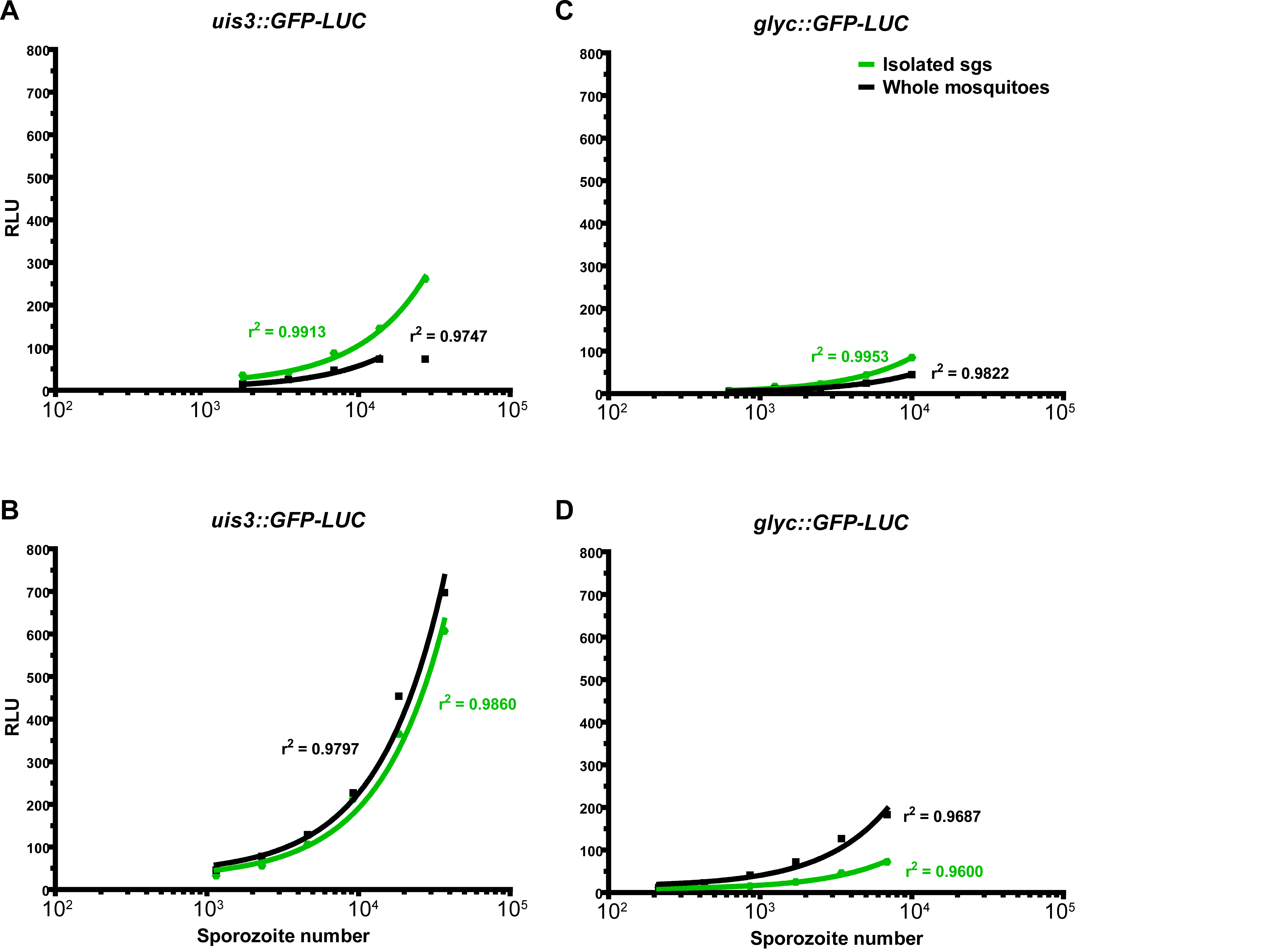

Supplement: Figure S1 — Correlation between sporozoite numbers and luciferase activity in sgs and in whole mosquitoes of uis3::GFP-LUC and glyc::GFP-LUC . Mosquitoes were infected with either uis3::GFP-LUC (A and B) or glyc::GFP-LUC (C and D) and sporozoites extracted from salivary glands or whole mosquitoes from the same experiment were collected 18–19 dpi. The sporozoites and whole mosquitoes were lysed and dilution series of cell extracts were generated and used to perform luciferase assays. Luciferase activity was measured and plotted as the value after subtraction of the baseline against the sporozoite number (RLU). Goodness of the linear curve fit is given as r2. 104 sgs correspond to 3.1 mosquito equivalents (A), 2.2 in (B), 6 in (C) and 2.1 in (D). In (A) reading of the highest sgs concentration for whole mosquitoes was excluded from the curve fit as an outlier. (TIF) [file pone.0036376.s001.tif]
