# Supplementary material for: Salivary Gland-Specific P. berghei Reporter Lines Enable Rapid Evaluation of Tissue-Specific Sporozoite Loads in Mosquitoes
Source: PLoS One. 2012 May 4;7(5):e36376. doi: 10.1371/journal.pone.0036376 (PMC3344870; doi:10.1371/journal.pone.0036376)
Supplement: Table S1 — Sporozoite numbers in dsTEP1 and dsLp mosquitoes. A. gambiae mosquitoes injected with dsRNA against TEP1(dsTEP1), Lp (dsLp) and LacZ (dsLacZ, control) were infected with PbGFPCON and salivary gland sporozoites were isolated from 7–18 mosquitoes 19–21 dpi to quantify sporozoite loads. (DOC) [file pone.0036376.s002.doc]

**Table S1.**

|  | ***dsLacZ*** | ***dsLp*** | ***dsTEP1*** |
| --- | --- | --- | --- |
| **Experiment 1** | 2916 | 685 | NA |
| **Experiment 2** | 1275 | 177 | NA |
| **Experiment 3** | 4000 | 1243 | 23422 |
